# Ameliorating effect of zinc on water transport in rice plants under saline-sodic stress

**DOI:** 10.3389/fpls.2025.1616333

**Published:** 2025-08-14

**Authors:** Kun Dang, Hao Tian, Jingjing Bai, Pengcheng Fu, Jiehao Cui, Dongming Ji, Xiwen Shao, Yanqiu Geng, Qiang Zhang, Liying Guo

**Affiliations:** ^1^ College of Agriculture, Jilin Agricultural University, Changchun, China; ^2^ Siping Agricultural Technology Extension Station, Siping, China; ^3^ Key Laboratory of Germplasm Innovation and Physiological Ecology of Dryland Food Crops, Ministry of Education, Harbin, China

**Keywords:** rice, saline-sodic stress, zinc, water transport, aquaporin

## Abstract

Saline-sodic stress not only impacts the absorption of nutrient ions, such as Zn^2+^, in rice but also induces physiological water shortages and ion toxicity in rice plants, significantly hindering their growth. To investigate this phenomenon, the present study utilized two rice varieties, ‘Changbai 9’ and ‘Tonghe 899’, as test subjects to simulate conditions of saline-sodic soil stress. Four-week-old rice seeds under four treatments: control (CT), 2 μmol L^-1^ zinc treatment alone (Z), 50 mmol L^−1^ saline-sodic treatment (S), and 50 mmol L^−1^ saline-sodic treatment with 2 μmol L^-1^ zinc (Z+S). The study aimed to examine the effect of zinc on water transport in rice plants under conditions of saline-sodic stress. Research indicates that the application of zinc positively influences the growth of rice under saline-sodic stress.The application of zinc not only reduces the Na^+^/K^+^ ratio and malondialdehyde (MDA) content, but also increases the levels of Zn^2+^, Cu^2+^, and other ions. Additionally, it enhances the expression of aquaporins in the plasma membrane of rice roots, which in turn increases the hydraulic conductance of the roots and ultimately improves the water absorption capacity of the root system under stress conditions. Additionally, zinc application promotes auxin (IAA) synthesis, facilitating root growth and expanding the root absorption area, which in turn enhances the water absorption rate and helps maintain higher leaf water content. Moreover, zinc application regulates stomatal conductance through an increase in potassium ion concentration and abscisic acid (ABA) content, thereby elevating the transpiration rate of rice leaves and promoting water absorption and transportation within the rice plants. Therefore, the addition of zinc under saline-sodic stress not only alleviates the effects of such stress but also enhances water absorption and transportation in rice plants. This results in a higher water content within the plants, positively influencing their growth and development under saline-sodic conditions.

## 1 Introduction

Various types of abiotic stresses present considerable threats to food production and impact crop yields globally (Zhou et al., 2023). In China, particularly in the northeast region, rice cultivation is a vital agricultural industry. However, soil salinization in northeast China significantly restricts rice production (Huang et al., 2022). Studies have demonstrated that crops will inevitably absorb Na^+^. When Na^+^ accumulate in crops to a certain level, they cause ion stress, disrupt the normal balance of Na^+^ and K^+^ within the plants, and consequently affect the physiological metabolism of the plants (Zörb et al., 2018). The adverse effects of salt on plant growth can be attributed to both osmotic stress and ion toxicity. The study found that high concentrations of salt ions in the external environment reduce the osmotic potential of the plant growth medium, thereby limiting water absorption and causing physiological water shortages in plants (Alkharabsheh et al., 2021). Concurrently, elevated salinity damages the root structure of crops, further diminishing their ability to absorb water (Shelden and Munns, 2023). Additionally, high pH levels in saline-sodic soils negatively impact respiration and nutrient uptake by crop roots (Zhang et al., 2016). Notably, the highly alkaline soil environment can inflict severe damage on the roots and young tissues of crops (Feng et al., 2023). Research has demonstrated that excessive Na^+^ can impede the absorption of Mg^2+^, Mn^2+^, and K^+^ ions by crops (Khan et al., 2008). In summary, saline-sodic stress adversely impacts crops in multiple ways, including the inhibition of growth and photosynthesis, as well as the disruption of water and nutrient absorption.

Zinc is an essential trace nutrient crucial for the normal growth and development of crops. It acts as a cofactor in various enzymatic reactions, including auxin and chlorophyll synthesis (Castiglione et al., 2023; Xian et al., 2024). Zinc not only promotes the growth of stem tips, young leaves, and roots but also facilitates the efficient progression of photosynthesis (Castiglione et al., 2023; Xian et al., 2024). Additionally, zinc plays a vital role in maintaining the redox (oxidation reduction) state of cells, serving as the catalytic center for various enzymes, such as superoxide dismutase and carbonic anhydrase. Carbonic anhydrase is particularly important in photosynthesis, as it accelerates the decomposition of dihydrocarbonic acid, thereby enhancing the absorption and utilization of released carbon dioxide by plants (Stanton et al., 2022). Furthermore, zinc enhances the resilience of crops against adverse environmental conditions, including improving their ability to withstand saline-sodic stress. Studies have demonstrated that saline-sodic stress can induce the production of a significant amount of reactive oxygen species in plant cells, leading to oxidative damage (Emami Bistgani et al., 2023). The application of zinc has been shown to enhance the activity of antioxidant enzymes in maize plants, thereby mitigating the effects of saline-sodic stress (Naseer et al., 2023). Our previous research indicated that zinc application increased the pigment content of rice leaves subjected to saline-sodic stress, improved the functionality of the PSII donor/acceptor side, and elevated the redox rate of PSI (Dang et al., 2024a). Furthermore, zinc application not only reduced the accumulation of soluble sugars and starch in rice leaves grown in saline-sodic soil but also alleviated the feedback inhibition of photosynthesis, facilitating the transport of assimilated products to the underground portions of the plant (Dang et al., 2024a). Several studies have demonstrated that zinc application can enhance root growth in crop cultivated in saline-sodic soil, leading to increases in root length, root volume, and root hair density, thereby improving plant adaptability to saline-sodic stress (Jorfi et al., 2022). Zinc is crucial for crop growth and development; under conditions of saline-sodic stress, zinc application can enhance the salt tolerance and antioxidant capacity of crops, as well as promote root growth, which helps protect crops from the detrimental effects of saline-sodic stress (Singh et al., 2024). Our previous research indicated that zinc application could improve carbohydrate metabolism in rice roots (Dang et al., 2024a). Therefore, further investigation is warranted to determine whether zinc can alleviate osmotic stress induced by saline-sodic conditions and promote water absorption in rice plants.

Tolerance to osmotic stress and the maintenance of optimal water status are critical components of plant salt tolerance (Singh et al., 2022). Our preliminary research indicates that zinc not only enhances the transpiration rate of rice seedling leaves under saline-sodic conditions but also increases the relative water content of these leaves (Dang et al., 2024b). This finding suggests that zinc may be instrumental in regulating water uptake in plants (Ali et al., 2021). The primary organ responsible for water absorption in rice is the root system, particularly the root hair zone at the root tip (Kim et al., 2020). Research has demonstrated that osmotic stress can alter the morphological structure of rice roots, subsequently impacting their water absorption capacity (Zhu et al., 2023). Under stress conditions, the root system may expand its water absorption area by increasing root length, root surface area, and other morphological traits, thereby enhancing water uptake (Li et al., 2023). Furthermore, root hydraulic conductivity is indicative of the root’s ability to absorb water and is influenced by factors such as driving force, root structure, and root water permeability (Fu et al., 2024). Notably, root water permeability can be modulated by aquaporins. Studies have shown that aquaporin activity and osmotic gradients are crucial in regulating root water uptake under stress conditions (Hachez et al., 2011). Additionally, phytohormones significantly influence water absorption and transport in rice, primarily by regulating stomatal movement and root growth (Ali et al., 2019; Bakaeva et al., 2022).

The osmotic gradient is a crucial driver of water uptake by roots. Root structure significantly influences the regulation of water uptake through the apoplastic pathway (Zhang et al., 2016). While the role of zinc in mitigating oxidative stress has been extensively documented in previous studies, its role in the osmotic regulation of root water uptake under saline-sodic stress remains less explored. Additionally, the regulatory effect of zinc on aquaporin expression in rice seedlings subjected to saline-sodic stress requires further investigation. Therefore, this study employed a NaCl: Na_2_SO_4_:Na_2_CO_3_:NaHCO_3_ ratio of 1:9:1:9 to simulate saline-sodic stress, examining the impact of exogenous zinc on sodium accumulation and root water absorption in rice under these conditions, and discussing the underlying mechanisms (Dang et al., 2024a). We hypothesized that: 1) Zinc can inhibit the absorption of Na^+^ by rice under saline-sodic stress while simultaneously promoting the absorption of K^+^. 2) Zinc promotes the growth of rice roots and enhances water absorption through the regulation of plant hormones. 3) Zinc improves water absorption in rice under saline-sodic stress by modulating aquaporin genes. 4) Zinc alleviates osmotic stress, mitigates physiological drought in rice under saline-sodic stress, and promotes overall growth and development. The application of zinc mitigates saline-sodic stress and enhances the capability of rice roots to absorb water and transport it to the leaves under such conditions. This intervention helps maintain the balance of water transport within rice plants, ultimately promoting their growth. This study aims to provide a theoretical basis for the high-yield and efficient cultivation of rice in saline-sodic areas.

## 2 Materials and methods

### 2.1 Experimental design

This study selected two rice varieties, ‘Changbai 9’ and ‘Tonghe 899’, as test materials, with the latter being more sensitive to saline-sodic stress. Uniform rice seeds were sterilized in a 5% sodium hypochlorite solution for 10 minutes. The seeds were then thoroughly rinsed with deionized water and germinated on gauze soaked in deionized water for 48 hours at a temperature of 28°C. Following germination, the seedlings were transferred to a 1/2 nutrient solution for 3 days and subsequently moved to a full nutrient solution for 3 weeks. Rice seedlings exhibiting consistent growth were selected for the subsequent treatments: control (CT), zinc alone treatment (Z), saline-sodic treatment (S), and saline-sodic plus zinc treatment (Z+S). Samples were collected for measurement after 7 days of saline-sodic and zinc treatment. The hydroponic experiment was conducted in an artificial climate culture room, utilizing a photoperiod of 14 hours of light at 28°C and 10 hours of darkness at 22°C, with the relative humidity maintained at approximately 70%. The nutrient solution was prepared based on the method for Hoagland nutrient solution, with slight modifications. The improved Hoagland full concentration nutrient solution consisted of 91.4 mg L^-1^ NH_4_NO_3_, 40.3 mg L^-1^ NaH_2_PO_4_·2H_2_O, 71.4 mg L^-1^ K_2_SO_4_, 88.6 mg L^-1^ CaCl_2_, 324 mg L^-1^ MgSO_4_·7H_2_O, 1.5 mg L^-1^ MnCl_2_·4H_2_O, 0.074 mg L^-1^ (NH_4_)_6_Mo_7_O_24_·4H_2_O, 0.934 mg L^-1^ H_3_BO_3_, 0.031 mg L^-1^ CuSO_4_·5H_2_O, 5.68 mg L^-1^ Na_2_SiO_3_.9H_2_O, 5.57 mg L^-1^FeSO_4_·7H_2_O (Gao et al., 2022). The nutrient solution was renewed every three days. To simulate saline-sodic, a mixture of NaCl, Na_2_SO_4_, Na_2_CO_3_, and NaHCO_3_ in a ratio of 1:9:1:9 was utilized, with a saline-sodic concentration of 50 mmol L^-1^ (the molar concentration is calculated as Na^+^, pH 8.5, and the conductivity is 3673 µS cm^-1^). Zinc sulfate heptahydrate was used as the zinc source at a concentration of 2 μmol L^-1^, which was determined to be optimal through previous concentration screening experiments. The pH of the nutrient solution was adjusted to 5.5 using either 0.2 M H_2_SO_4_ or 1 M KOH every two days.

HgCl_2_ acts as an inhibitor of aquaporin, while DTT has the capacity to counteract the inhibitory effects of HgCl_2_ (Knipfer et al., 2011). The rice seedlings subjected to a 7 d treatment of saline-sodic, along with a combined treatment of saline-sodic and zinc, were categorized into three distinct groups: CK treatment (without HgCl_2_), HgCl_2_ treatment, and DTT treatment. In the HgCl_2_ treatment, HgCl_2_ (50 μM) was added to the culture medium after the 7 d saline-sodic stress, thoroughly mixed, and allowed to react for 5 min. Following this, the root system was rinsed with distilled water and placed back into a solution devoid of HgCl_2_ for rapid determination of transpiration rate. In the DTT treatment, after the same 7 d saline-sodic stress, the root system was exposed to HgCl_2_ (50 μM), mixed thoroughly, and treated for 5 min. It was then rinsed with distilled water and placed in a solution containing 5 mM DTT to restore aquaporin activity, after which the transpiration rate was rapidly determined.

### 2.2 Sampling and determination

#### 2.2.1 Determination of Na^+^ and K^+^ contents in rice leaves and roots

After seven days of treatment with saline-sodic and zinc, three rice plants exhibiting similar growth across the treatments were selected. The leaves and roots of these plants were excised and subjected to drying at 105°C for 30 minutes, followed by drying at 80°C. The dried samples were then weighed, ground into a fine powder, and passed through a 100 mesh screen (Ma et al., 2020). A 0.5 g accurately weighed sample was subsequently heated and digested using H_2_SO_4_-H_2_O_2_. Using a flame photometer (M410, Sherwood Science LTD., Cambridge, UK), the power was activated, and a gasoline combustion pump was engaged to adjust the intake speed, generating a zigzag flame upon ignition. The boiled sample was then analyzed, and the readings were recorded (Wang et al., 2022). The concentrations of Na^+^ and K^+^ in the sample were calculated based on the standard curve generated (Zhang et al., 2020).

#### 2.2.2 Determination of malondialdehyde content and relative electrolyte leakage rate

After seven days of saline-sodic and zinc treatment, three rice plants exhibiting consistent growth across the treatments were selected. Their leaves and roots were excised to measure the malondialdehyde (MDA) content and the relative electrolyte leakage rate (REL) (Bao et al., 2017). The content of MDA content was determined using the thiobarbituric acid colorimetric method. Specifically, 1000 g of rice leaves were weighed and ground, then mixed with 10 mL of 10% trichloroacetic acid (TCA) to create a homogenate (Giridharan et al., 2020). The homogenate was centrifuged at 6000×g for 20 minutes. Subsequently, 1 mL of the supernatant was added to 2 mL of a reaction solution and boiled for 15 minutes. The reaction solution consisted of 0.6% (v/v) thiobarbituric acid (TBA) and 10% (w/v) trichloroacetic acid. Centrifuge the sample again at 4000xg for 15 minutes. Collect the supernatant and measure the absorbance values at 450, 532, and 600 nm. Lipid peroxidation levels were quantified in nanomoles per gram of fresh weight, utilizing an extinction coefficient of 155 mM^-1^cm^-1^. The relative electrolyte leakage (REL) of rice leaves was determined according to the method described by Dionisio-Sese and Tobita. Fresh leaves weighing 1.000 g were washed with deionized water and then transferred to test tubes containing 15 ml of deionized water. The leaves were incubated at room temperature (25°C) for 2 hours, after which the electrical conductivity (E_1_) was measured using a conductivity meter (DS-307, Shanghai Reitz, Shanghai, China). Subsequently, the test tube was placed in an environment of 100°C for 30 minutes and then cooled back to room temperature (25°C) to measure the conductivity (E_2_). The relative electrolyte leakage rate (REL) is calculated as follows:


(1) $$\text{REL}=\frac{{\text{E}}_{1}}{{\text{E}}_{2}}\times 100\%$$

#### 2.2.3 Determination of gas exchange parameters

On the 0th, 1st, 3rd, 5th, and 7th days of saline-sodic and zinc treatment, a portable photosynthetic measurement system (Li-6400, Li-Cor Inc, USA) was employed to assess the photosynthetic performance of rice plants subjected to different treatments between 9:00 and 11:00 AM. The leaves were expanded to measure the net photosynthetic rate (Pn), stomatal conductance (Gs), and intercellular carbon dioxide concentration. Each treatment was replicated three times. During the measurements, the leaf chamber temperature was maintained at approximately 26°C, with a light intensity of 800 μmol·m^-2^·s^-1^, a CO_2_ concentration of 400 μmol mol^-1^, and relative humidity ranging from 60% to 70%. Additionally, the transpiration rates of rice leaves subjected to HgCl_2_ and DTT treatments were measured.

#### 2.2.4 Determination of nutrient ion concentration

After 7 days of saline-sodic and zinc treatment, three rice plants exhibiting consistent growth across treatments were selected. Their leaves and root systems were subjected to thermal destruction at 105°C for 30 minutes, followed by drying at 80°C. The samples were then weighed, ground into a fine powder, and processed through a 100 mesh sieve. Following the method of Wu et al (Wu et al., 2021). with slight modifications, 0.1 g of the sample that had passed through a 2 mm sieve was accurately weighed and placed into a digestion tank. Subsequently, 3 mL of HNO_3_ and 1 mL of HF were added, and digestion was performed using a microwave digestion instrument (MD20H model, Chengdu Opule Instrument Co., Ltd., China). Once the sample was digested to a clear and transparent state, an electric graphite acid remover (GD25/GD40 model, Chengdu Opule Instrument Co., Ltd., China) was utilized to catch the acid. The resulting solution was then diluted with ultrapure water and the volume adjusted to 100 mL. The contents of Si, Mn, Fe, Mg, Cu, and Zn in the plants were measured using an inductively coupled plasma optical emission spectrometer (ICP-1000II, Beijing Haowei Technology Co., Ltd., China). Each treatment was conducted in triplicate.

#### 2.2.5 Determination of water status of leaves

Samples were collected at 0, 1, 3, 5, and 7 days following saline-sodic and zinc treatments, with three seedlings included in each treatment group. Bound water content (BWC) and free water content (FWC) were assessed using fresh samples. RWC and total water content (TWC) were measured following the methods outlined by MaChado and Paulsen (2001), while FWC was determined according to the protocol established by Ming et al. (2011). Fresh leaves were promptly rinsed with distilled water, weighed, and recorded as fresh weight (FW), then dried and immersed in a 60% sucrose solution for 6 hours. This procedure was conducted in a dark environment at 4°C. Subsequently, the leaves were swiftly removed, washed with ultrapure water (at least three times), dried to remove surface moisture, weighed, and recorded as stem weight (SW). Finally, the leaves were dried at 70°C until a constant weight was achieved, weighed, and recorded as dry weight (DW). Calculations were performed according to the following formula:


(2) FWC(%)=(FW−SW)/DW×100

(3) BWC(%)=TW−FWC

#### 2.2.6 Determination of plant hormones

After 7 days of saline-sodic and zinc treatment, three rice plants exhibiting consistent growth across treatments were selected. Their leaves and roots were immediately frozen in liquid nitrogen and subsequently stored at -80°C for the analysis of auxin, abscisic acid, and salicylic acid. All measurements were conducted using kits produced by Shanghai Enzyme Biotechnology Co., Ltd. (Shanghai, China) and were performed in three biological replicates. The concentrations of abscisic acid (ABA), indole-3-acetic acid (IAA), and salicylic acid (SA) in plant tissues were quantified using a one-step sandwich enzyme-linked immunosorbent assay (ELISA) employing double antibodies. Initially, the plant tissues were ground into a fine powder. An appropriate volume of extract was subsequently added, and the mixture was shaken for a specified duration. Following this, the supernatant was obtained through centrifugation. The ABA, IAA, and SA present in the supernatant were allowed to interact with specific antibodies, resulting in the formation of antigen-antibody complexes. These complexes were then reacted with an enzyme-labeled substrate, leading to a color change. Finally, the intensity of the color change in the reaction solution was measured using colorimetry, and the concentration of ABA, IAA, and SA was determined based on a standard curve (He et al., 2015).

#### 2.2.7 Root structure scanning

After seven days of saline-sodic and zinc treatment, three rice plants exhibiting consistent growth across the treatments were selected. The root system structure, including surface area and diameter, was then scanned using a root system scanner.

#### 2.2.8 Determination of active root absorption area

After 7 days of saline-sodic and zinc treatment, select three rice plants exhibiting consistent growth across the treatments. Rinse the roots briefly with distilled water and gently absorb excess moisture using absorbent paper. Measure the root volume employing the drainage method. Subsequently, immerse the roots in three test tubes containing an equal volume of methylene blue solution (0.075 mg mL^-1^) for 1.5 minutes. Immediately after soaking, remove the roots and allow the methylene blue solution to drain until no further solution drips from them. From each of the three test tubes, take 1 ml of the methylene blue solution, dilute it tenfold with distilled water, and measure the absorbance at 660 nm. Concurrently, prepare a standard curve using the methylene blue solution to calculate the active absorption area of the root system per unit volume based on the standard curve and the measured root volume (Li X. et al., 2016).

#### 2.2.9 Root water uptake rate

After seven days of saline-sodic and zinc treatment, three rice plants exhibiting consistent growth across the treatments were selected to measure the water absorption rate of the rice roots (Gupta et al., 2016). The gravity method was employed for this measurement. Two days prior to the measurement, the seedlings were transplanted into 500 ml plastic bottles, each sealed at the mouth with one seedling per bottle. Measurements were conducted between 8:00 AM and 12:00 PM. The weight of each bottle was recorded every two hours, with a control bottle containing no plants for comparison. The root water absorption rate was calculated based on the weight difference before and after the measurement.

#### 2.2.10 Determination of the osmotic potential of rice root xylem sap

The osmotic potential of xylem sap was measured following the methodology described by Yin et al. (2013). Rice seedlings were cut 4 cm from the base of the roots, and the entire root system was placed into a pressure chamber, ensuring that the incision exposed the sealing ring for several centimeters. After sealing the chamber, a small amount of pressure was applied to induce the flow of xylem fluid from the incision, which was subsequently collected. The osmotic potential of the wound fluid was measured using an osmotic potential meter (Model 5520, Wescor, Logan, UT, USA). The penetration potential was calculated using the formula: ψs = -RTC, where R (the gas constant) is 8.314×10^-6^(MPa L mmol^-1^ K^-1^), T is the temperature in Kelvin, and C (mmol kg^-1^) represents the reading obtained from the instrument, indicating the concentration of the isotonic solution.

#### 2.2.11 Determination of soluble sugar content in roots.

After 7 days of saline-sodic and zinc treatment, three rice plants exhibiting consistent growth across treatments were selected. Their leaves and roots were harvested and subjected to a killing treatment at 105°C for 30 minutes, followed by drying at 80°C. The dried samples were then weighed and ground into fine powder, which was passed through a 100 mesh sieve. The determination of root soluble sugar was performed according to the method described by Wu et al. (2013) (Wu et al., 2013). Specifically, 1 g of the fresh sample was weighed, and 4 ml of 80% (v/v) ethanol was added. The mixture was extracted at 80°C for 40 minutes, followed by centrifugation at 2000 g for 15 minutes. The residue was extracted three additional times, and the supernatants were combined for the determination of soluble sugar. Sampling occurred on days 5, 10, and 15 of treatment, with three seedlings per treatment and three repetitions.

#### 2.2.12 Expression analysis of aquaporin gene

After 7 days of saline-sodic and zinc treatment, leaf and root samples from rice seedlings were collected, immediately frozen in liquid nitrogen, and stored at -80°C. RNA extracted and purified during sequencing was converted into cDNA using the cDNA Synthesis SuperMix for qPCR (One-Step gDNA Removal) kit from Beijing Quanshijin Biotechnology Co., Ltd. Subsequently, RT-qPCR was conducted using the PerfectStart^®^ Green qPCR SuperMix kit on the QuantStudio™ 3 Real-Time PCR System. The parameters included a denaturation phase (94°C for 30 s, followed by 94°C for 5 s and 60°C for 34 s) and an annealing and extension phase (95°C for 15 s, 60°C for 1 min, and 95°C for 15 s), repeated for a total of 40 cycles. The algorithm for quantitative expression analysis was based on the 2^-ΔΔCT^ method. The primer sequences for the internal reference gene and the *OsPIP* gene used in qPCR are referenced in **Table 1**. Each sample and internal reference gene (ACTIN) was analyzed in three replicates.

**Table 1:** The primers of the *OsPIP* genes and reference gene.

Gnen	Primer sequence	Gnen	Primer sequence
*OsPIP1;1*	*F: ATCTTCTGGGTTGGTCCCTTCGTT* *R: ATCACGATTGCGTTGCATGTCGTC*	*OsPIP2;4*	*F: CAACAACAACAAGGCCTGGAGTGA* *R: GAAAGAGCCCAAACAATGCCGACT*
*OsPIP1;2*	*F: ATGCCTGGGATGACCATTGGATCT* *R: ATGCAGGTTACGACCTGCTCTTGA*	*OsPIP2;6*	*F: CCTGGTTGGACTTGGTCATATCGT* *R: ACAAATCATGCACCTGGCTGACTG*
*OsPIP2;1*	*F: GCTGGAAGGCGTTGATGAAGCAAT* *R: ACTTCACACACACGACAAGCAGGA*	*ACT*	*F:GGGTTCACAAGTCTGCCTATTGT* *R: ACGGGACACGACCAAGGA*
*OsPIP2;2*	*F: CCCAATTGGATTCGCGGTGTTCAT* *R: ATCCCAGGCCTTGTCCTTGTTGTA*	*—*	*—*

### 2.3 Statistical analyses

Data were collected and analyzed using Microsoft Excel 2019 software. Following this, the data were further analyzed with the SPSS statistical software package version 22 (IBM Corp., Armonk, NY, USA). Descriptive statistical methods were employed to assess the mean values and standard errors of the measured parameters. This study utilized one-way analysis of variance (ANOVA), incorporating Duncan’s multiple comparison method. A statistically significant difference was established when p < 0.05. Results are presented as standard error (SE). The charts were generated using Origin 2021 software.

## 3 Results

### 3.1 The effects of zinc on the growth, dynamic water content, and gas exchange parameters of rice under saline-sodic stress

**Figure 1:**
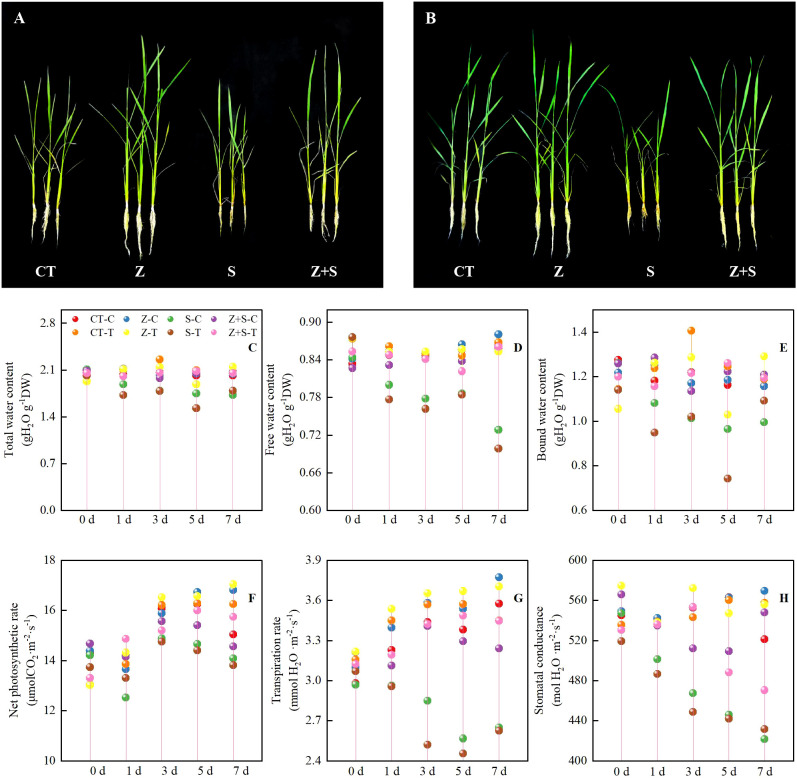
The effects of zinc on the growth state (**A:** Growth of ‘Changbai 9’, **B:** The growth of ‘Tonghe 899’), dynamic water content (**C:**Total water content, **D:** Free water content, **E:** Bound water content), and gas exchange parameters (**F:** Net photosynthetic rate, **G:** Transpiration rate, **H:** Stomatal conductance) of rice subjected to saline-sodic stress. The mean values of three repetitions ± SE (n=3) were used. CT, no saline-sodic and no zinc treatment; Z, zinc treatment; S, saline-sodic treatment; Z+S, saline-sodic and zinc treatment. C, ‘Changbai 9’ rice; T, ‘Tonghe 899’ rice.

**Figures 1A**, **B** illustrate that the growth trends of the two rice varieties are consistent across treatments. Specifically, saline-sodic stress significantly impacts rice growth, resulting in an increase in yellow leaves and shorter plants. The application of zinc mitigates these effects by reducing the number of yellow leaves and increasing plant height; however, the growth remains inferior compared to rice treated with zinc under non-stress conditions. Meanwhile, By measuring the dynamic water content (**Figures 1C–E**) and dynamic phosgene exchange parameters (**Figures 1F–H**) of rice leaves under various treatments, we observed that, under abiotic stress conditions, the water content and dynamic phosgene exchange parameters of rice leaves treated with CT and zinc increased over time, peaking on the 7th day. In contrast, as the duration of saline-sodic stress increased, both the moisture content and gas exchange parameters of rice decreased, reaching their lowest point on the seventh day. The application of zinc was found to mitigate the effects of saline-sodic stress, enhancing the moisture content of rice and improving its gas exchange parameters.

### 3.2 Effects of zinc on Na^+^, K^+^, MDA content and REL in rice leaves and roots under saline-sodic stress

**Figure 2:**
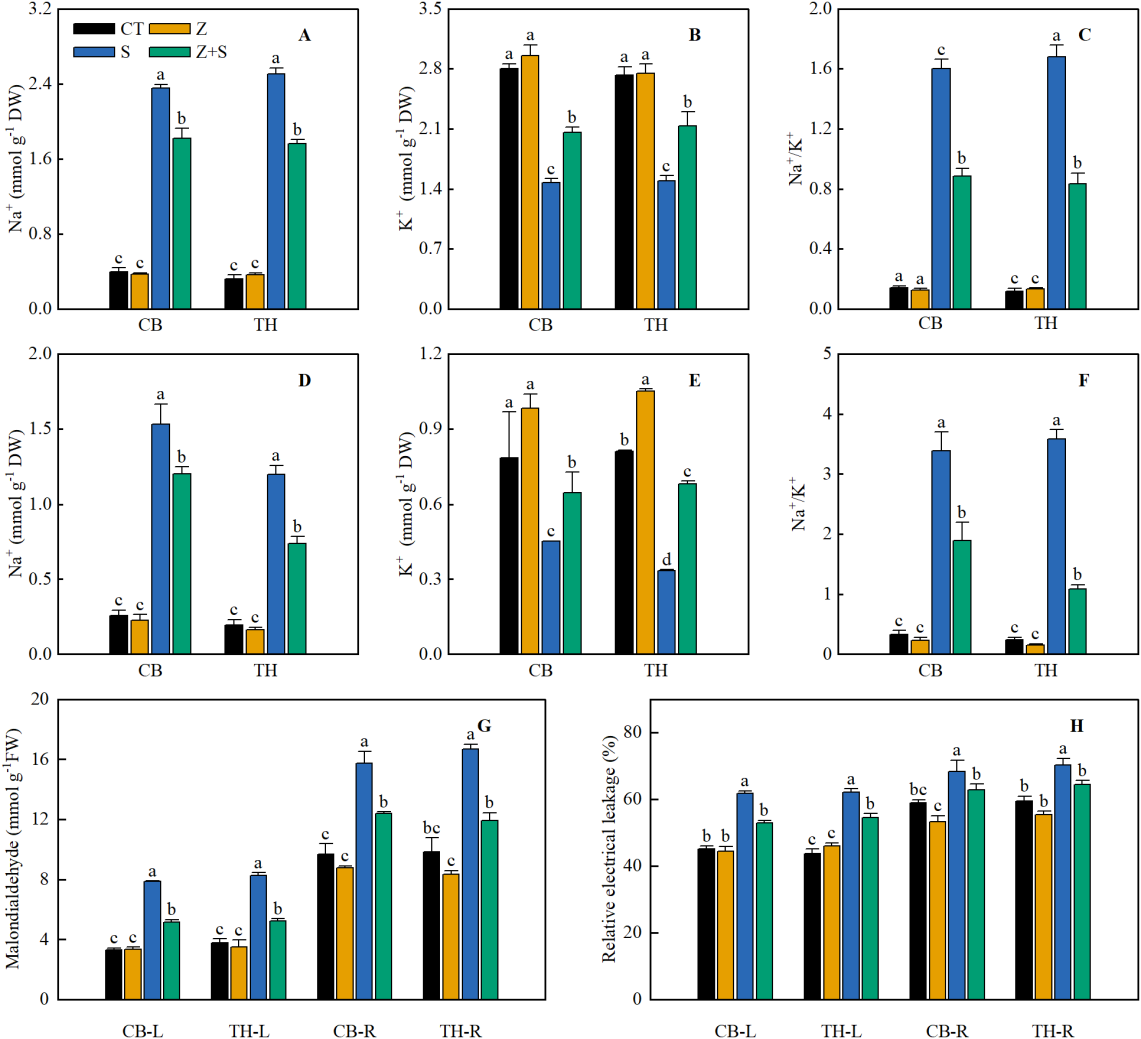
This study investigates the effects of zinc on the concentrations of Na^+^ **(A, D)**, K^+^ **(B, E)**, malondialdehyde (MDA) **(G)**, Na^+^/K^+^ **(C, F)**, and relative electrolyte leakage (REL) **(H)** in the leaves **(A-C)** and roots **(D-F)** of two rice varieties subjected to saline-sodic stress. The mean values of three repetitions ± SE (n=3) were used. CT, no saline-sodic and no zinc treatment; Z, zinc treatment; S, saline-sodic treatment; Z+S, saline-sodic and zinc treatment. CB-L, ‘Changbai 9’ rice leaves; TH-L, ‘Tonghe 899’ rice leaves; CB-R, ‘Changbai 9’ rice root; TH-R, ‘Tonghe 899’ rice root.

**Figure 2** illustrates that the effects of zinc on Na^+^, K^+^, and MDA contents, as well as REL in the leaves of two rice varieties under saline-sodic stress, are consistent. Under non-stress conditions, zinc application did not significantly affect the Na^+^, K^+^, and MDA contents and REL in the leaves of either rice variety. Conversely, saline-sodic stress markedly increased Na^+^ content, Na^+^/K^+^ ratio, MDA content, and REL value in the rice leaves, while decreasing K^+^ concentration. Zinc application had significant positive effects on the Na^+^ content, K^+^ concentration, Na^+^/K^+^ ratio, MDA content, and REL value of rice grown under saline-sodic stress. Specifically, the application of zinc markedly reduced the levels of Na^+^ and MDA, as well as the Na^+^/K^+^ ratio and REL values, while simultaneously increasing the K^+^ content. Compared to saline-sodic treatment alone, the combined treatment of saline-sodic and zinc resulted in a reduction of Na^+^ content, MDA content, and REL value in the leaves of the ‘Changbai 9’ rice variety by 22.8%, 34.6%, and 12.3%, respectively, while the K^+^ concentration increased by 12.3%. In the ‘Tonghe 899’ rice variety, the Na^+^ content, MDA content, and REL value of the leaves decreased by 29.6%, 36.7%, and 14.3%, respectively, with a corresponding increase in K^+^ concentration of 14.3%. Similarly, in the root system, the Na^+^ content, MDA content, and REL value of the ‘Changbai 9’ rice variety decreased by 21.6%, 21.4%, and 8.1%, respectively, while the K^+^ concentration increased significantly by 43.2%. For the ‘Tonghe 899’ rice variety, the Na^+^ content, MDA content, and REL value in the roots decreased by 38.4%, 28.6%, and 8.5%, respectively, accompanied by a substantial increase in K^+^ concentration of 104%.

### 3.3 Effects of zinc on nutrient ion content in rice leaves and roots under saline-sodic stress

**Figure 3:**
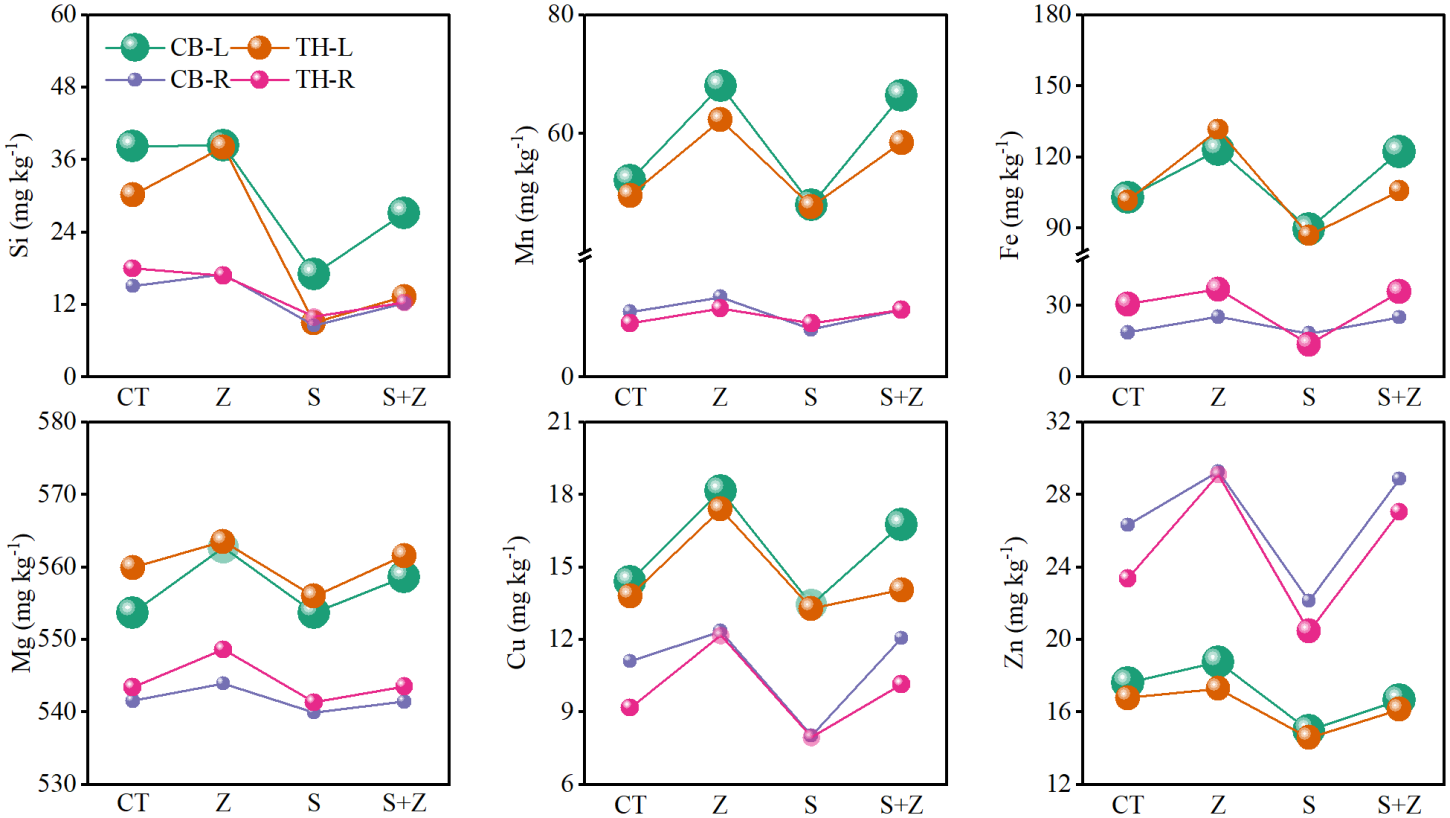
The effects of zinc on the nutrient ion content in the leaves and roots of two rice varieties under saline-sodic stress. CT, no saline-sodic and no zinc treatment; Z, zinc treatment; S, saline-sodic treatment; Z+S, saline-sodic and zinc treatment. CB-L, ‘Changbai 9’ rice leaves; TH-L, ‘Tonghe 899’ rice leaves; CB-R, ‘Changbai 9’ rice root; TH-R, ‘Tonghe 899’ rice root.

**Figure 3** illustrates the impact of zinc on nutrient ions in both the leaves and roots of rice, with a consistent trend observed across the two varieties. Specifically, under normal growth conditions, the application of zinc increased the concentrations of Si^2+^, Mn^2+^, Fe^2+^, Mg^2+^, Cu^2+^, and Zn^2+^ in the leaves and roots of both rice varieties. Conversely, saline-sodic stress resulted in a decrease in the concentrations of these nutrient ions in the leaves and roots. Notably, with the application of zinc, the concentrations of these ions in the leaves and roots of both rice varieties were enhanced. Therefore, the application of zinc proves beneficial for the absorption of nutrient ions in rice.

### 3.4 Effects of zinc on root morphology and vitality of rice under saline-sodic stress

**Figure 4:**
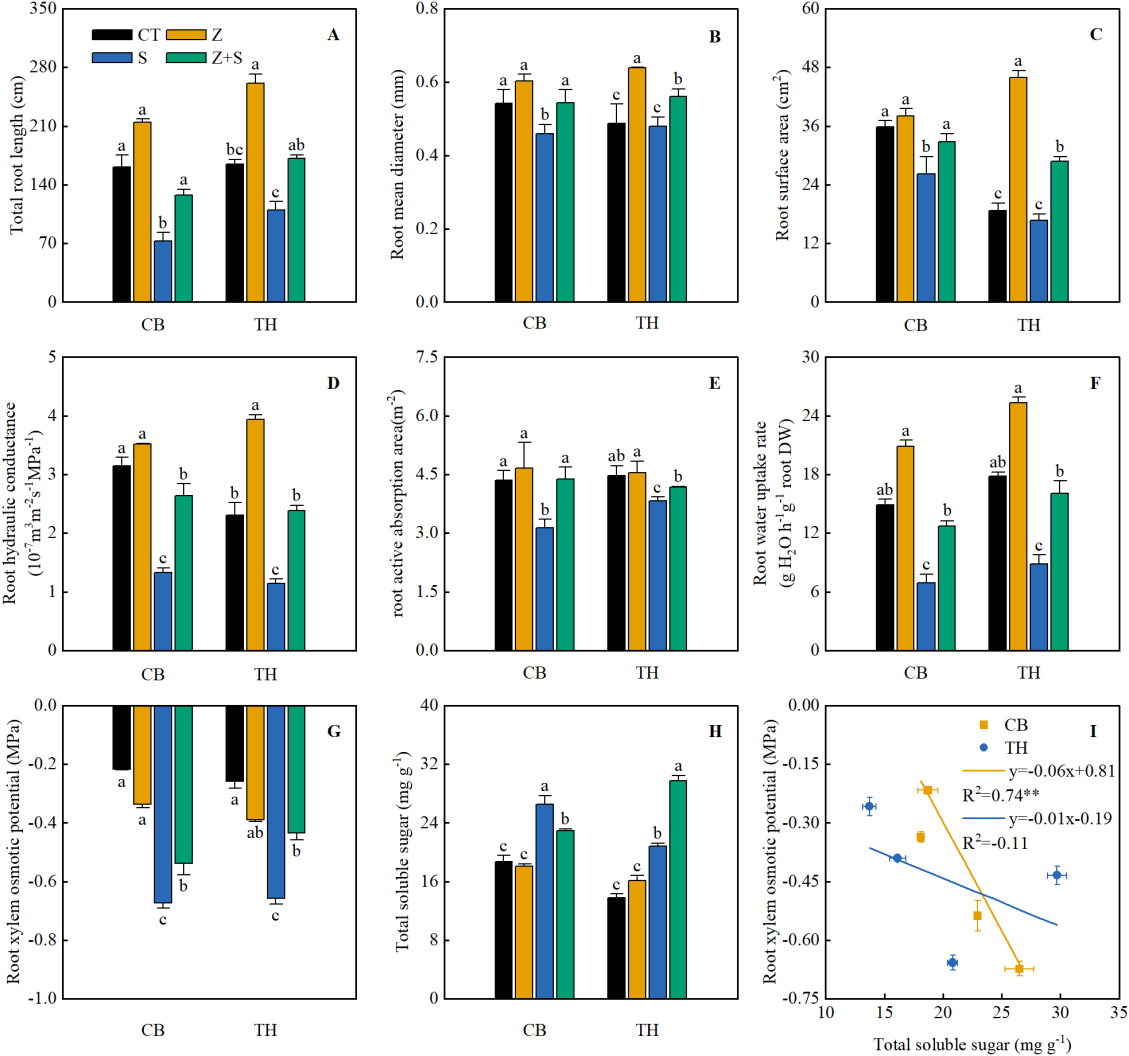
The effects of zinc on various root characteristics, including total root length **(A)**, root diameter **(B)**, root surface area **(C)**, root hydraulic conductivity **(D)**, root water absorption area **(E)**, root water absorption rate **(F)**, xylem penetration type **(G)** and total soluble sugar content **(H)** in the roots of two rice varieties subjected under saline-sodic stress. **(I)** illustrates the correlation analysis between root xylem penetration potential and total soluble sugar content. The mean values of three repetitions ± SE (n=3) were used, and different letters were used to indicate statistical significance at the p < 0.05 level. CT, no saline-sodic and no zinc treatment; Z, zinc treatment; S, saline-sodic treatment; Z+S, saline-sodic and zinc treatment.

As shown in **Figure 4**, under normal growth conditions, zinc application did not affect the total root length (RL), root diameter (RD), root surface area (RS), root hydraulic conductance (RHC), or root active absorption area (RAA) of the two rice varieties. However, saline-sodic stress treatment resulted in a decrease in total RL, RD, RS, RHC, and RAA for both rice varieties. Notably, when zinc was applied in conjunction with saline-sodic treatment, there was a significant increase in total RL, RD, RS, RHC, and RAA compared to the saline-sodic treatment alone. Under normal growth conditions, the application of zinc does not significantly affect the content of rice root xylem osmotic potential (RXOP) or total soluble sugar (TSS). However, saline-sodic stress markedly reduces RXOP and significantly increases TSS in the two rice varieties studied. In conditions of saline-sodic stress, zinc application enhances the RXOP of the roots in both rice varieties and increases the TSS content in ‘Tonghe 899’, while it decreases TSS in the roots of ‘Changbai 9’. Furthermore, as illustrated in **Figure 4I**, a significant negative correlation exists between the RXOP of the ‘Changbai 9’ rice variety and the TSS content in its root system. In contrast, No significant correlation was observed between RXOP and the TSS content of ‘Tonghe 899’.

### 3.5 Effect of zinc on gene expression of aquaporin in rice under saline-sodic stress

**Figure 5:**
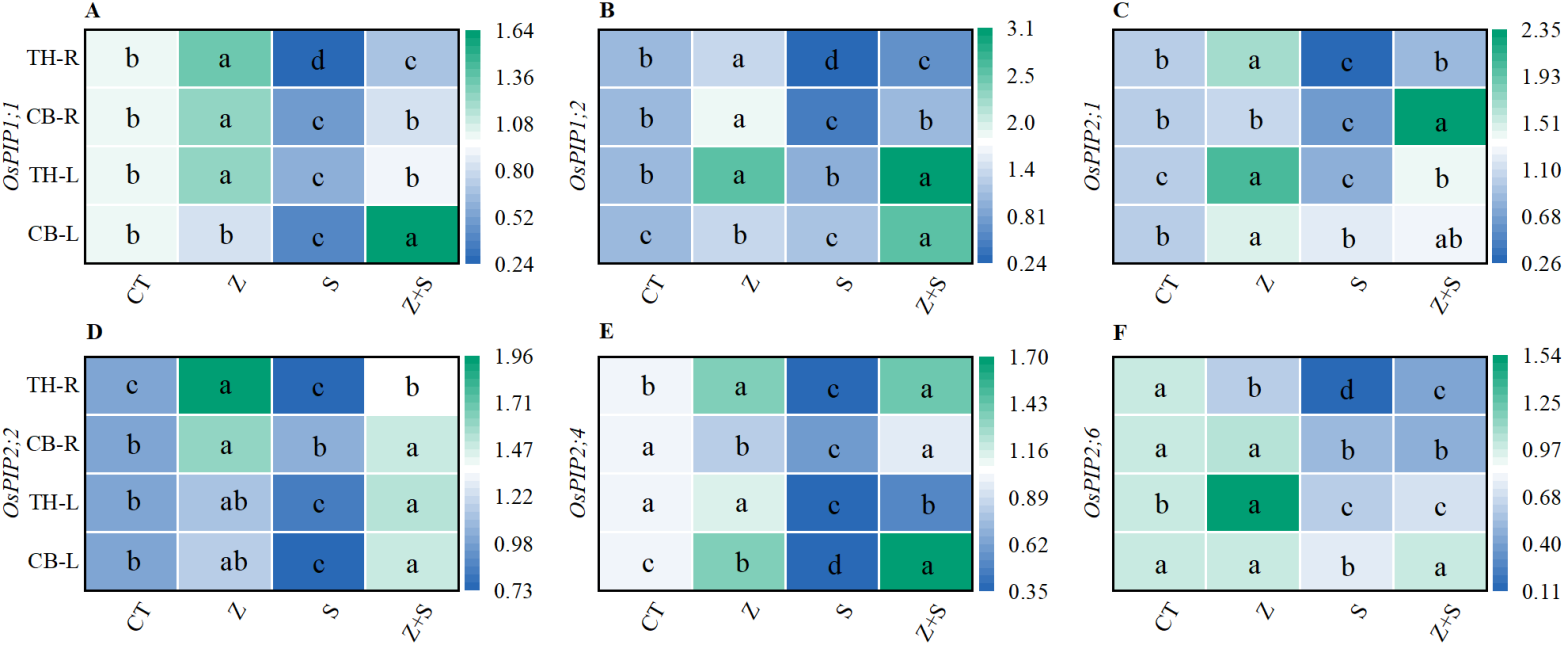
The effects of zinc on the aquaporin genes *OsPIP1;1*, *OsPIP1;2*, *OsPIP2;1*, *OsPIP2;2*, *OsPIP2;4*, and *OsPIP2;6* in the leaves and roots of rice under under saline-sodic stress. The mean values of three repetitions were used, and different letters were used to indicate statistical significance at the p < 0.05 level. CT, no saline-sodic and no zinc treatment; Z, zinc treatment; S, saline-sodic treatment; Z+S, saline-sodic and zinc treatment. CB-L, ‘Changbai 9’ rice leaves; TH-L, Tonghe 899 rice leaves; CB-R, ‘Changbai 9’ rice root; TH-R, ‘Tonghe 899’ rice root.

We utilized quantitative PCR to assess the expression of major plasma membrane aquaporins in rice roots. The results indicated that saline-sodic treatment resulted in a significant decline in the expression levels of the aquaporins *Os PIP1;1*, *Os PIP1;2*, *Os PIP2;1*, *Os PIP2;2*, *Os PIP2;4*, and *Os PIP2;6* in both the leaves and roots of the two rice varieties (**Figure 5**). Conversely, the addition of zinc significantly enhanced the expression levels of *Os PIP1;1*, *Os PIP1;2*, *Os PIP2;2*, and *Os PIP2;4*. In comparison to saline-sodic stress alone, the addition of zinc did not significantly alter the expression of *Os PIP2;1* in the leaves of ‘Changbai 9’ (**Figure 5C**), nor did it affect *Os PIP2;6* in the roots and *Os PIP2;6* in the leaves of ‘Tonghe 899’ (**Figure 5F**). However, the expression levels of *Os PIP2;1* in the leaves of ‘Tonghe 899’ and *Os PIP2;6* in the roots were increased with the addition of zinc. Notably, there was a significant effect on the expression levels of *Os PIP2;6* and *Os PIP2;6* (**Figure 5F**) in the leaves of ‘Changbai 9’.

### 3.6 Effects of zinc on the contents of auxin, abscisic acid and salicylic acid in rice leaves and roots under saline-sodic stress

**Figure 6:**
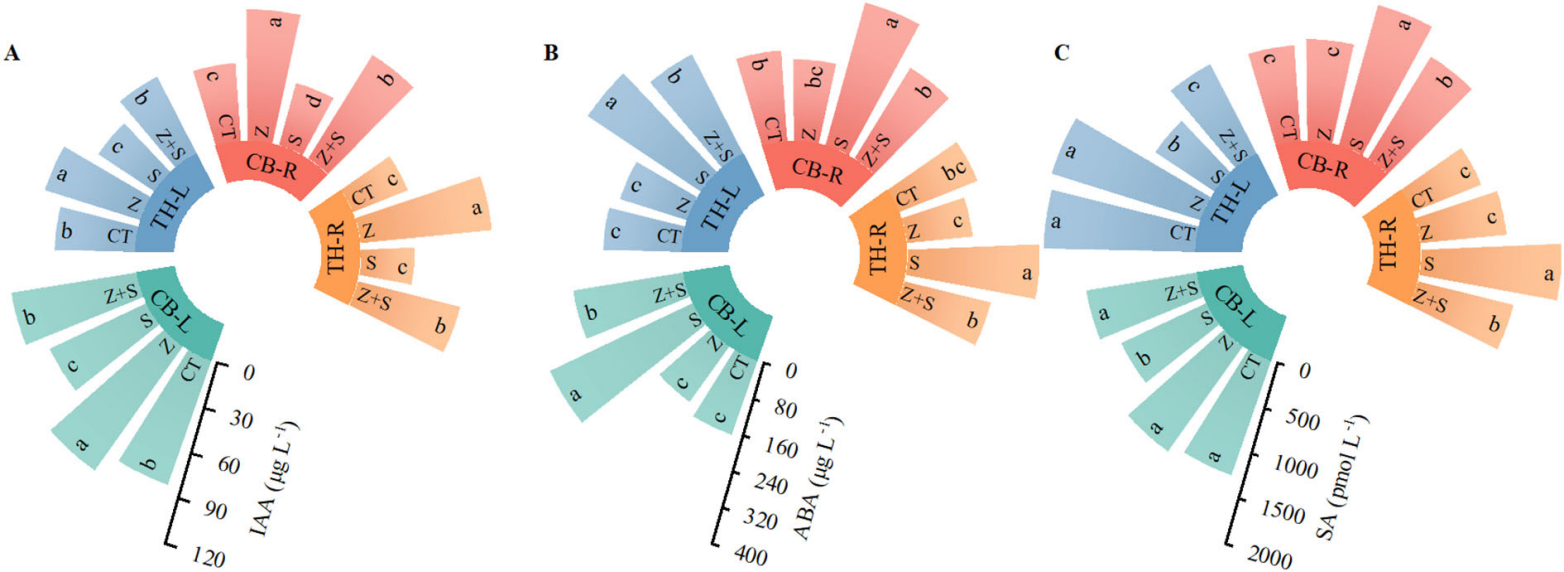
The effects of zinc on the concentrations of auxin **(A)**, abscisic acid **(B)**, and salicylic acid **(C)** in the leaves and roots of rice under saline-sodic stress. The mean values of three repetitions were used, and different letters were used to indicate statistical significance at the p < 0.05 level. CT, no saline-sodic and no zinc treatment; Z, zinc treatment; S, saline-sodic treatment; Z+S, saline-sodic and zinc treatment. CB-L, ‘Changbai 9’ rice leaves; TH-L, ‘Tonghe 899’ rice leaves; CB-R, ‘Changbai 9’ rice root; TH-R, ‘Tonghe 899’ rice root.

**Figure 6** illustrates the effects of zinc on the concentrations of auxin, abscisic acid, and salicylic acid in the leaves and roots of rice during the experimental treatment. Under non-stress conditions, zinc application significantly increased the levels of auxin (IAA) in both the leaves and roots of two rice varieties, while the contents of abscisic acid (ABA) and salicylic acid (SA) showed no significant changes. In contrast, saline-sodic stress significantly reduced auxin levels in the leaves and roots of both rice varieties, while increasing the concentrations of ABA and SA. However, zinc application under these stress conditions significantly enhanced auxin levels in the leaves and roots of the two rice varieties. It also led to a reduction in the concentrations of ABA and SA.

### 3.7 The relationship between ion content, cell membrane permeability, and plant hormones in the leaves and roots of rice under conditions of saline-sodic stress

**Figure 7:**
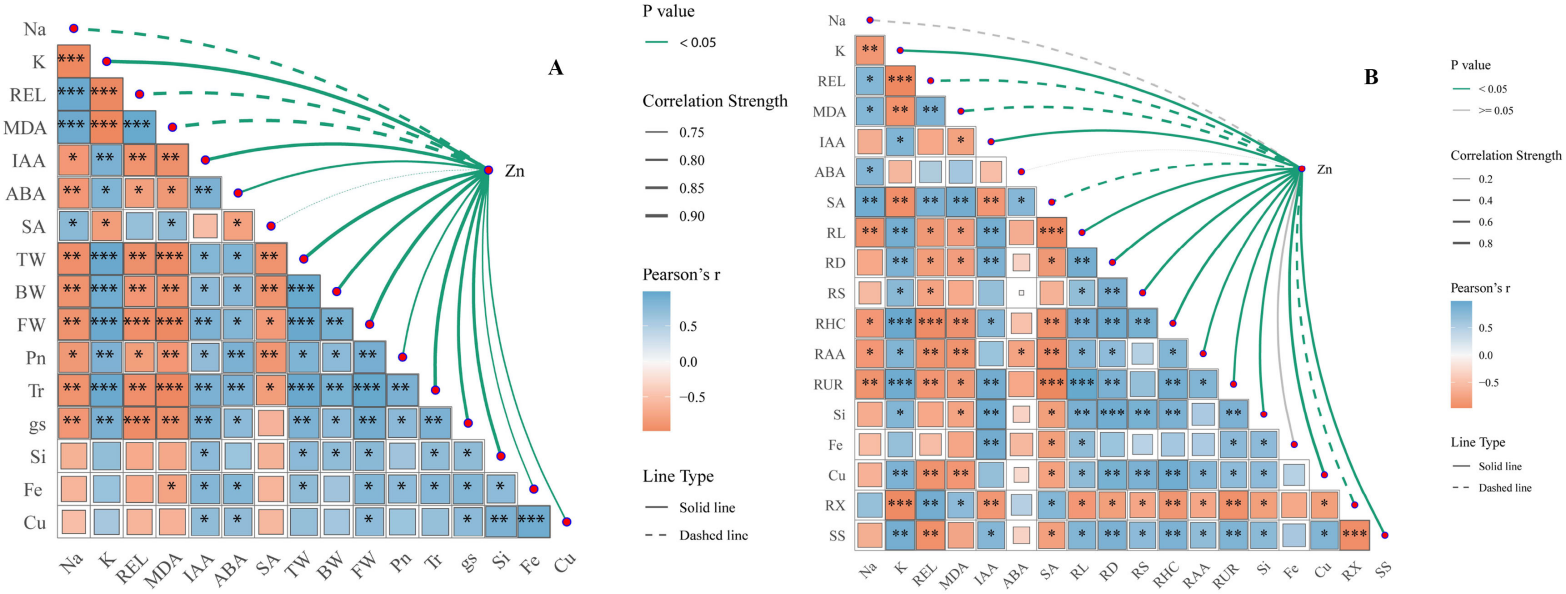
The correlation analysis of leaf ion content, gas exchange parameters, leaf water content, and leaf hormones **(A)**, as well as root morphology, ion content, root water absorption, and root hormones **(B)**. Asterisks (*) indicate significant correlations at P < 0.05. Abbreviations used include: REL for relative electricity leakage, MDA, malondialdehyde; IAA, auxin; ABA, abscisic acid; SA, salicylic acid; TW, total water content; BW, bound water; FW, free water; Tr, Transpiration rate; Pn, net photosynthetic rate; g_s_, stomatal conductance; RL, total root length; RD, root diameter; RS, root surface area; RHC, root hydraulic conductivity; RAA, root water absorption area; and RUR, root water absorption rate.

**Figure 7A** illustrates the correlation between Zn and Na^+^ content, cell membrane permeability, and plant hormones in rice leaves. Zinc exhibits a significant negative correlation with Na^+^, MDA, REL, and SA. Conversely, Zn shows a significant positive correlation with K^+^, IAA, ABA, TW, BW, FW, Pn,Tr, gs, Si, Fe, and Cu. **Figure 7B** illustrates the correlation between Zn and Na^+^ content, cell membrane permeability, root morphology, and plant hormones in rice roots. The results indicate that Zn is significantly negatively correlated with Na^+^, MDA, REL, ABA, and SA. Conversely, Zn exhibits a positive correlation with K^+^, IAA, ABA, RL, RD, RS, RHC, RAA, RUR, Si, Fe, and Cu. Therefore, The application of zinc reduced the Na^+^ content in rice leaves and roots, alleviated saline-sodic stress, and ensured the integrity of the cell membrane. Additionally, it increased the K^+^ and IAA content in both rice leaves and roots, which not only enhanced g_s_ but also promoted root growth, thereby benefiting water absorption and trans port. Ultimately, the growth and photosynthesis of rice under saline-sodic stress were improved.

### 3.8 Correlations between ion content in leaves and roots, membrane permeability, and plant hormones under conditions of saline-sodic stress

**Figure 8:**
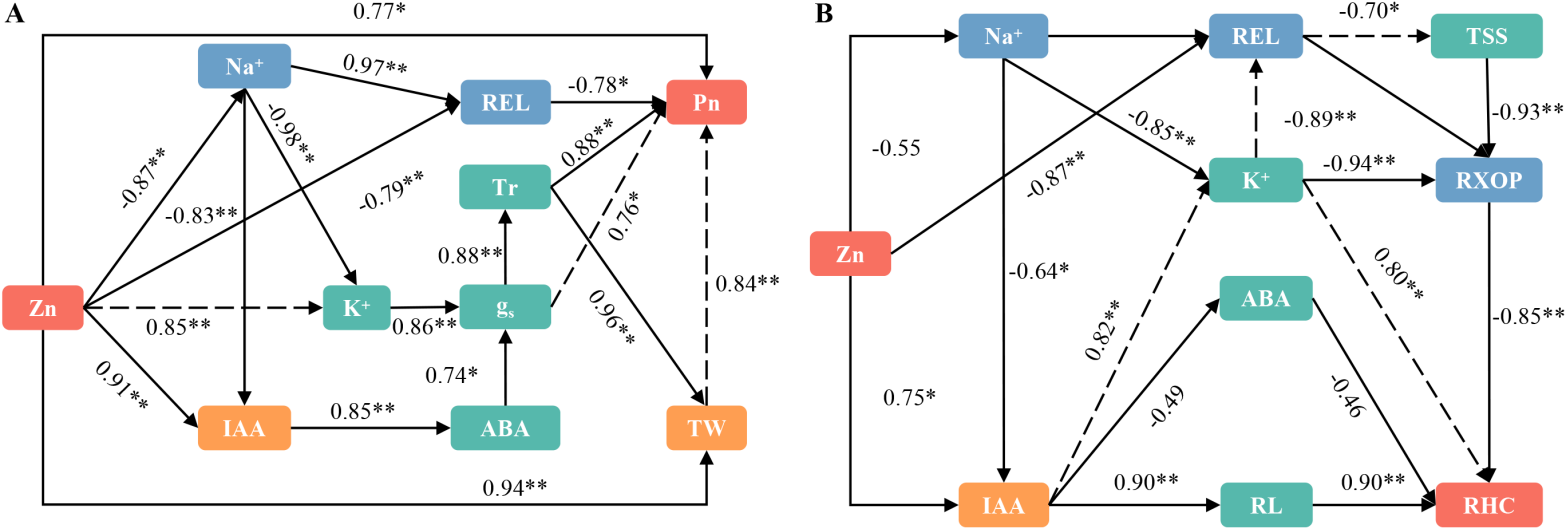
Presents a structural equation model (SEM) that illustrates the relationships among various factors in rice leaves **(A)** and roots **(B)**. The significance levels are indicated as *P < 0.05, **P < 0.01, and ***P < 0.001. REL, relative electricity leakage; MDA, malondialdehyde; IAA, auxin; ABA, abscisic acid; TW, total water content; Tr, Transpiration rate; Pn, net photosynthetic rate; gs, stomatal conductance; RL, total root length; RHC, root hydraulic conductivity; TSS, Total soluble sugar; RXOP, Root xylem penetration potential; AG, Aquaporin gene.

As shown in **Figure 8A**, the application of zinc directly reduced the Na^+^ content in rice leaves and decreased the relative permeability of the cell membrane. This reduction was beneficial for the normal functioning of photosynthesis in rice leaves. Concurrently, the application of zinc increased the levels of K^+^ and IAA, which directly regulated the content of abscisic acid. This interplay resulted in a significant increase in g_s_ due to the co-regulation of K^+^ and ABA. Consequently, the Tr increased, enhancing the water content of rice leaves and promoting photosynthesis. **Figure 8B** illustrates the correlation between ion content in roots, membrane permeability, and plant hormones. The application of zinc demonstrates a direct effect on Na^+^ content, while not significantly altering it; however, it does directly influence REL. This alteration subsequently affects the xylem permeability of rice roots, thereby enhancing water absorption. Additionally, zinc application significantly increases IAA content, which promotes the growth of rice roots and facilitates water absorption. Furthermore, zinc directly regulates the water channel protein gene, further promoting water uptake by rice roots. In conclusion, the application of zinc has significant positive effects on both water absorption and transport in rice.

### 3.9 The effects of HgCl_2_ and DTT on the transpiration rate of rice seedlings

**Figure 9:**
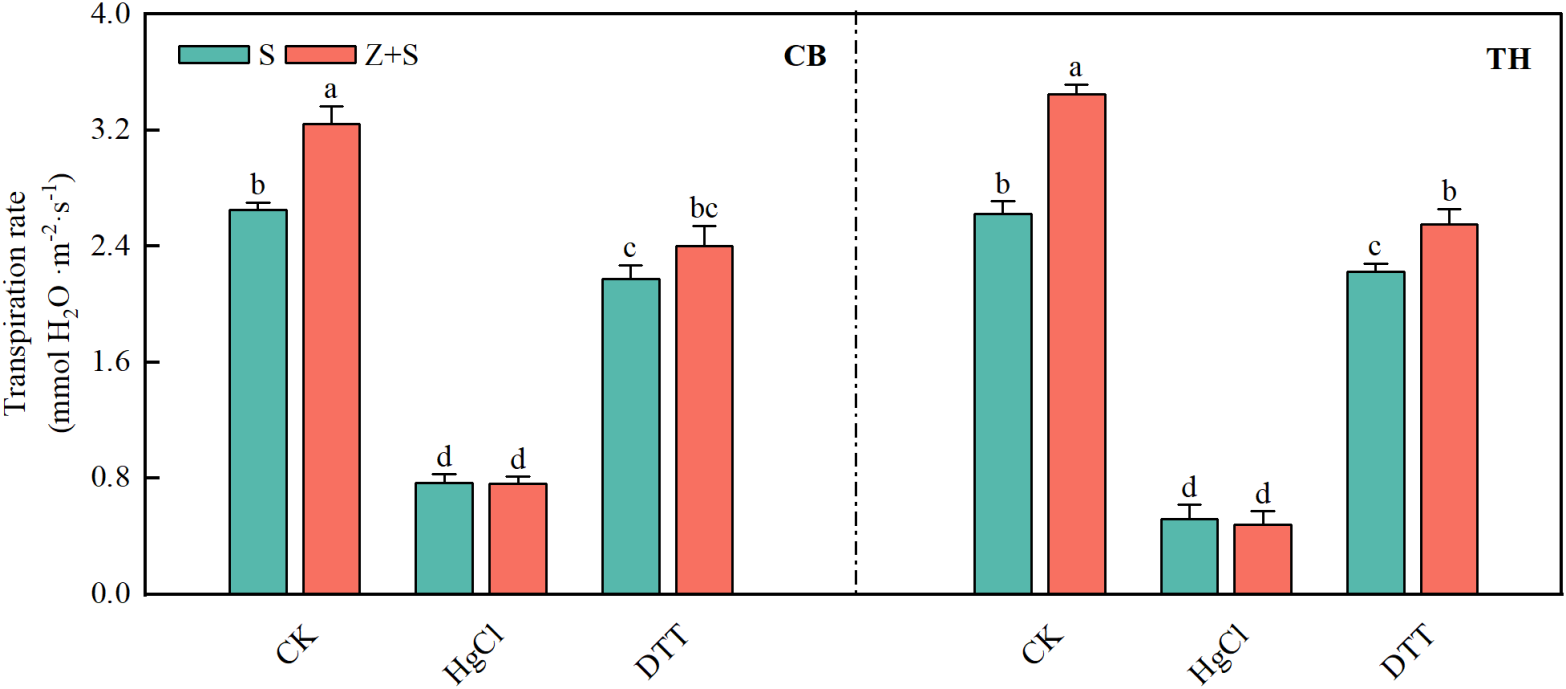
the effects of mercuric chloride (HgCl_2_) and dithiothreitol (DTT) on the transpiration rate of rice leaves subjected to saline-sodic stress and zinc treatment. The mean values of three repetitions were used, and different letters were used to indicate statistical significance at the p < 0.05 level. S, saline-sodic treatment; Z+S, saline-sodic and zinc treatment. CB, ‘Changbai 9’ rice; TH, ‘Tonghe 899’ rice.

As illustrated in **Figure 9**, The changes observed in the two rice varieties are fundamentally similar. zinc enhances the transpiration rate of rice leaves subjected to saline-sodic stress. Furthermore, following HgCl_2_ treatment, the transpiration rate of rice seedlings exhibited a significant decline, leading to the loss of distinction between the S and Z+S treatments. Upon recovery from DTT treatment, the transpiration rates of both S and Z+S treatments increased. however, they remained lower than the levels observed prior to HgCl_2_ treatment. Notably, the transpiration rate in the zinc-added treatment continued to exceed that of the saline-sodic treatment alone.

## 4 Discussion

The elevated concentration of salt ions and the high pH levels characteristic of saline-sodic land adversely impact the absorption of essential nutrient ions, such as zinc, by rice (Li Y. et al., 2016). Additionally, these conditions lead to physiological water shortages, ion toxicity, and oxidative damage in plants, significantly hindering their growth (Wang et al., 2019). In this study, we investigated the ameliorative effects of zinc on rice subjected to saline-sodic stress by assessing various parameters, including rice growth, the photosynthetic system, ion balance, hormone levels, and the regulation of water transport through aquaporins. Our findings indicate that zinc enhances the salt stress resistance of rice, facilitates water absorption and transport, and promotes overall growth and development under saline-sodic stress.

### 4.1 Relieving effect of zinc on ion toxicity of rice under saline-sodic stress

Saline-sodic stress significantly impacts the growth and development of rice in the northeastern region of my country (Zhou et al., 2022). Research indicates that under saline-sodic stress, a substantial accumulation of Na^+^ occurs in rice, leading to ion toxicity, disrupting the normal sodium and potassium balance, and adversely affecting physiological metabolic functions (Zhang L. et al., 2021). The increase in relative electrolyte leakage (REL) and malondialdehyde (MDA) content in crop plants serves as an indicator of both the extent of salt damage and the salt tolerance of these crops. Research has demonstrated that saline-sodic stress leads to the absorption of significant amounts of Na^+^ by rice, promoting the accumulation of malondialdehyde in the leaves (Khan et al., 2022). This process exacerbates cell membrane lipid peroxidation and enhances REL, aligning with the findings of this study. As illustrated in **Figure 2**, saline-sodic stress markedly increased Na^+^ concentrations in the leaves and roots of both rice varieties. Concurrently, the values for MDA and REL also rose. Notably, the application of zinc in this study positively influenced the reduction of Na^+^, MDA content, and REL values in the leaves and roots of rice under saline-sodic stress. This effect may be attributed to the facilitation of Zn^2+^ absorption in rice, where zinc, being a crucial component of the cell membrane, helps maintain membrane integrity and enhances resistance to saline-sodic stress (Vermeulen et al., 2019). As illustrated in **Figures 7** and **8**, the zinc content in rice leaves and roots exhibits a significant negative correlation with both Na^+^ content and REL values. Furthermore, zinc has the ability to directly reduce the Na^+^ content and REL in rice leaves. Under saline-sodic stress conditions, Na^+^ primarily traverses through non-selective cation channels and high-affinity K^+^ channels. The similar hydration radii of Na^+^ and K^+^ result in competition for binding sites on a substantial number of Na^+^ and K^+^ channels, consequently inhibiting K^+^ absorption (Bose et al., 2013; Guo et al., 2021). In this study, the application of zinc was found to limit the absorption of Na^+^ by rice under saline-sodic stress, resulting in a reduced concentration of Na^+^ in rice tissues (**Figures 2A, D**) while simultaneously enhancing the absorption of K^+^ (**Figures 2B, E**). Furthermore, this study demonstrated that zinc application significantly elevated the Cu^2+^ content in rice leaves and roots, which in turn enhanced the activity of the antioxidant enzyme superoxide dismutase (SOD), promoted the decomposition of reactive oxygen species (ROS), and reduced the accumulation of ROS. This reduction ultimately mitigated the damage to cell membranes caused by ROS accumulation.

### 4.2 Improving effect of zinc on root growth and water transport of rice under saline-sodic stress

Under saline-sodic stress, it is crucial for plants to maintain a balance between water absorption and loss (Pan et al., 2016). Sustaining a higher water content during stress and enhancing the water absorption rate of the root system can effectively dilute salts within the plant, thereby improving its ability to resist saline-sodic stress (Xu et al., 2021). In plants, the limitation of water transport primarily occurs in the roots (Shi et al., 2016). Root anatomy and surface characteristics play a crucial role in regulating water uptake through the apoplastic pathway (Liu et al., 2014). This study demonstrates that saline-sodic stress significantly restricts the growth of rice roots; however, the application of zinc proves beneficial for root development. As illustrated in **Figures 4A–C**, the application of zinc markedly enhances the growth rate of rice roots under saline-sodic stress, increasing total root length, root surface area, and root diameter. This improvement is attributed to the promotion of auxin (IAA) synthesis in rice roots due to zinc application (**Figure 6B**), which subsequently fosters root growth. Zinc, as a vital element in IAA synthesis, can directly influence rice roots in the context of saline-sodic stress, as indicated by the IAA content shown in **Figure 8B**. Root hydraulic conductivity reflects the water absorption capacity of the root system and is influenced by factors such as the driving force, root anatomy, root surface characteristics, and root water permeability (Zhao et al., 2021). Root hydraulic conductance serves as a crucial indicator of the root system’s ability to absorb and transport water. Previous studies have demonstrated that enhancing root hydraulic conductivity can significantly increase the water absorption rate of roots, which aligns with the findings of this study (Geng et al., 2018). As illustrated in **Figure 7B**, saline-sodic stress adversely impacts the hydraulic conductance of rice roots, consequently diminishing their water absorption rate. Additionally, saline-sodic stress inhibits the growth of rice roots and reduces the moisture absorption area. The application of zinc not only promotes the growth of rice roots but also increases the water absorption area and hydraulic conductance, thereby enhancing the overall water absorption rate of rice. Studies have demonstrated that root hydraulic conductivity is primarily influenced by aquaporins (Schneider et al., 2020). This research further corroborates this finding. Specifically, the study revealed that saline-sodic stress significantly decreased the expression levels of *OsPIP1;1*, *OsPIP1;2*, *OsPIP2;1*, *OsPIP2;2*, *OsPIP2;4*, and *OsPIP2;6* in the leaves and roots of two rice varieties (**Figure 5**). The addition of zinc significantly increased the expression levels of *OsPIP1;1*, *OsPIP1;2*, *OsPIP2;2*, and *OsPIP2;4*. However, this study also found that zinc supplementation did not significantly alter the expression of *OsPIP2;1* in the leaves of ‘Changbai 9’ (**Figure 5C**) or *OsPIP2;6* in the roots (**Figure 5F**), nor did it affect the expression of *OsPIP2;6* in the leaves of ‘Tonghe 899’ (**Figure 5F**). Conversely, it significantly affected the expression levels of *Os PIP2;1* in the leaves of ‘Tonghe 899’ (**Figure 5C**) and *Os PIP2;6* in the roots (**Figure 5F**), as well as *Os PIP2;6* in the leaves of ‘Changbai 9’ (**Figure 5F**), suggesting that the regulatory effect of zinc on aquaporins may vary among rice varieties. To investigate the role of aquaporin in water absorption, we employed the aquaporin inhibitor HgCl_2_ for further verification (**Figure 9**). The results confirmed that aquaporin is involved in water absorption in rice. Additionally, the enhancement of water absorption in rice roots under saline-sodic stress, facilitated by zinc, is partly mediated through the regulatory effects of aquaporin. In conclusion, the results of this experiment demonstrate that under saline-sodic stress, zinc can enhance the expression of aquaporins in the plasma membrane of rice roots, thereby promoting root hydraulic conductivity and ultimately improving the water absorption capacity of roots under stress conditions.

Osmotic adjustment ability is a fundamental characteristic of plants that enables them to resist saline-sodic stress (Mao et al., 2012). Through osmotic regulation, plant cells can lower their intracellular water potential, thereby enhancing their capacity for water absorption (Barragán et al., 2012). This study demonstrated that saline-sodic stress significantly increased the total soluble sugar content in two rice varieties. Notably, zinc application led to a significant reduction in the soluble sugar content of the roots of ‘Changbai 9’, while it continuously increased the soluble sugar content in the roots of ‘Tonghe 899’. Furthermore, saline-sodic stress markedly decreased the root xylem sap osmotic potential in both rice varieties, whereas zinc application significantly enhanced the root xylem sap osmotic potential (**Figure 4G**). The reduction in the osmotic potential of xylem sap in rice roots facilitates water absorption by the roots (Osakabe et al., 2013). In this study, the xylem osmotic potential of the root system of ‘Changbai 9’ exhibited a significant negative correlation with the soluble sugar content of the root system (**Figure 4I**). Conversely, the continuous increase in soluble sugar within the root system of ‘Tonghe 899’ did not impact the xylem osmotic potential of rice roots. These findings suggest the presence of interspecific and intraspecific variations in osmotic regulation effects among the varieties. Additionally, it is plausible that the increase in other osmotic regulatory substances, such as ions and soluble proteins, in the root system of ‘Tonghe 899’ contributed to the regulation of the xylem osmotic potential in rice roots.

### 4.3 Regulation of zinc on photosynthesis in rice under saline-sodic stress

Studies have demonstrated that saline-sodic stress leads to an increase in excessive salt ions, which in turn decreases osmotic potential, adversely affects root water absorption, induces stomatal closure, and diminishes photosynthesis and transpiration capabilities (Hasanuzzaman et al., 2021). These effects result in osmotic stress for plants, ultimately culminating in water deficits (Pan et al., 2021). This study further corroborates that saline-sodic stress can significantly reduce the stomatal conductance of rice leaves and inhibit the transpiration rate. Notably, the application of zinc enhanced stomatal conductance (**Figure 1H**), which subsequently increased the transpiration rate (**Figure 1G**). Stomata serve as the primary channels for water and gas exchange between plants and their environment (Li et al., 2022). The size of stomata, along with the CO_2_ concentration gradient both inside and outside the leaves, directly influences the transpiration and photosynthesis of plants (Guo et al., 2022). The enhancement of stomatal pores in rice under saline-sodic stress due to zinc may be linked to the accumulation of K in rice leaves (Zhang Q. et al., 2021). Numerous studies have demonstrated that K^+^ plays a vital role not only in osmotic regulation but also in stomatal regulation (Yan et al., 2018). The increase in stomatal conductance is closely associated with the significant role of abscisic acid (ABA) in stomatal control (Nouraei et al., 2022). The interaction between IAA and ABA plays a pivotal role in regulating root development and stomatal function, two critical processes that determine plant adaptation to environmental stresses. IAA, the primary auxin in higher plants, is essential for root architecture, particularly lateral root initiation (LRI) and elongation, while ABA is a key hormone in mediating responses to abiotic stresses such as drought and salinity (Emenecker and Strader, 2020). In arabidopsis thaliana, the cyclic nucleotide-gated ion channel gene AtCNGC4 mutant (dnd2) exhibits elevated levels of both IAA and ABA, which leads to altered stomatal regulation and enhanced drought tolerance. The dnd2 mutant demonstrates increased stomatal conductance under ABA treatment, indicating that IAA may modulate ABA’s effect on stomatal closure (Kale et al., 2019). These findings underscore the complex interplay between IAA and ABA in optimizing stomatal behavior, a crucial adaptation for water conservation in variable environments. In this study, the application of zinc significantly increased the IAA content, promoted the growth of rice roots, reduced ABA levels, and influenced the stomatal closure of rice leaves. Furthermore, the transpiration rate is a key driver of water uptake and transport within plants (Zhang et al., 2022). This study demonstrates that the increase in stomatal conductance directly influences the transpiration rate of rice leaves (**Figure 8A**), thereby enhancing water absorption and transport within rice plants. Concurrently, the application of zinc also facilitates the absorption of silicon in both rice leaves and roots (**Figure 4**), which further contributes to the elevation of the transpiration rate in rice leaves. It is noteworthy that while the application of zinc can enhance the stomatal conductance and transpiration rate of rice leaves, it does not decrease the water content of these leaves. This suggests that zinc primarily improves the water status of the aboveground parts of rice by enhancing root water absorption. Additionally, the improvement in the water status of rice leaves may be associated with increased stem hydraulic conductivity (Klepsch et al., 2018). However, further research is required to elucidate the effects of zinc on stem hydraulic conductivity and its role in improving the water status of rice under saline-sodic stress.

In summary, saline-sodic stress not only impairs the absorption of nutrient ions, such as zinc, in rice but also leads to physiological water shortages, ion toxicity, oxidative damage, and other detrimental effects on plant health, thereby significantly hindering plant growth (Jiang et al., 2020). However, the application of zinc has been shown to positively influence the growth of rice under saline-sodic stress. The application of zinc not only reduces the Na^+^/K^+^ ratio and malondialdehyde (MDA) content but also increases the levels of Zn^2+^, Cu^2+^, and other ions. This dual effect helps mitigate damage to the cell membrane caused by reactive oxygen species (ROS) accumulation, thereby preserving the integrity of the cell membrane. Zinc enhances the expression of aquaporins in the plasma membrane of rice roots, which increases hydraulic conductance and ultimately improves the water absorption capacity of the roots under stress conditions. The application of zinc is advantageous for the synthesis of auxin, which in turn promotes the growth of rice roots. This enhancement increases the absorption area of the roots, elevates the water absorption rate, and supports the maintenance of higher leaf water content. Furthermore, zinc application regulates stomatal conductance by elevating potassium ion concentration and ABA levels, thereby increasing the transpiration rate of rice leaves and facilitating water absorption and transport within the plants.

## 5 Conclusion

Under saline-sodic stress, it is crucial for plants to maintain a balance between water absorption and loss, as well as to sustain a higher water content to enhance their resistance to such stress. This study demonstrates that the addition of zinc under salt stress can improve the hydraulic conductivity of rice roots and their water absorption capacity by modulating the expression of aquaporin genes. However, the regulation of aquaporin expression is not the sole mechanism through which zinc enhances root water absorption. Zinc also plays a significant role in regulating plant antioxidant capacity and osmotic balance, which further supports root water absorption. Moreover, zinc promotes plant growth and stomatal regulation by influencing rice plant hormones, thereby improving water transport in rice plants subjected to saline-sodic stress and maintaining an overall high-water content. In summary, the addition of zinc under saline-sodic stress not only alleviates the impacts of such stress but also promotes water absorption and transport in rice plants, maintains elevated water content, and positively influences the growth and development of rice under these challenging conditions.

## Data Availability

The original contributions presented in the study are included in the article/supplementary material. Further inquiries can be directed to the corresponding authors.
